# Incidence, demographics, and survival of patients with primary pituitary tumors: a SEER database study in 2004–2016

**DOI:** 10.1038/s41598-021-94658-8

**Published:** 2021-07-26

**Authors:** Cheng Chen, Yu Hu, Liang Lyu, Senlin Yin, Yang Yu, Shu Jiang, Peizhi Zhou

**Affiliations:** 1 Department of Neurosurgery, West China Hospital of Sichuan University, No. 37, Guoxue Alley, Chengdu, 610041 Sichuan Province People’s Republic of China; 2 State Key Laboratory of Biotherapy, Sichuan University, Chengdu, Sichuan Province People’s Republic of China; 3 Pituitary Adenoma Multidisciplinary Center, West China Hospital of Sichuan University, Chengdu, Sichuan Province People’s Republic of China

**Keywords:** Neuroendocrine diseases, Epidemiology

## Abstract

Comprehensive investigations on the incidence and prognosis of pituitary tumors are still lacking. The present study aims to summarize the incidence, demographics, and survival outcome of pituitary adenoma on a population-based level. This study includes all pituitary adenomas reported in the Surveillance, Epidemiology, and End Results (SEER) database from 2004 to 2016 in the United States. Extensive clinical and demographic characteristics were extracted and submitted to group comparisons. The standardized incidence rate was calculated and stratified by year at diagnosis, age/sex and age/treatment groups. The Kaplan–Meier analysis and multivariable regressions were performed to identify the factors associated with overall survival. A total of 47,180 pituitary tumors were identified, including 47,030 typical adenomas, 111 uncertain behavior pituitary adenomas, and 39 pituitary carcinomas. The overall standardized incidence rate was 4.8 cases per 100,000 person-years and the annual incidence rate continually trended upwards, with a peak seen in 2015. We noticed a bimodal age-related distribution in females and a unimodal distribution in males. In the multivariate regression analysis, the factors associated with prolonged survival included typical adenoma, younger age, and smaller tumor size. Whereas, black and male patients had worse overall survival. Our study provides a reliable estimate on the incidence of pituitary adenoma and confirms that the annual standardized incidence rate is increasing. Pituitary adenomas have a satisfactory long-term prognosis and age, tumor size, and tumor subtypes are related to overall survival. Though statistically significant, our inferential findings should be constrained within the limitations of SEER database.

## Introduction

With a prevalence of approximately 1/1000 in case-finding studies, pituitary tumors are more frequent than thought^1^. Although the etiology of pituitary tumors covers a wide range of pathologies, most of them are typical benign adenomas. Despite their generally benign nature, these tumors can lead to considerable morbidity and elevated mortality^2–6^, which pose a significant burden to health care resources. A comprehensive investigation regarding the incidence and long-term prognosis of primary pituitary adenoma is crucial to better understand the natural course of the disease and lend support to the medical decision-making process. However, robust population-based estimates relating to the incidence and overall survival of pituitary tumors are still lacking and the existing data of epidemiological characteristics are discordant^6–10^.

On January 1, 2004, cancer registries in the Surveillance, Epidemiology, and End Results (SEER) project began to identify and abstract benign and borderline tumors of the central nervous system^11^. The collection of benign brain tumors provided us with enormous opportunities for disease epidemiology analysis. Therefore, our present study aims to summarize the incidence rate, demographics, and survival outcome of all primary pituitary tumors diagnosed between 2004 and 2016 in the SEER database.

## Materials and methods

### Patient selection

The cohort utilized in the current research was extracted from the SEER 18 registries customized database (with additional treatment fields), which covers approximately 27.8% of the US population and with an appropriate representation of most ethnicities in the United States^12^. After signing the Research Data Agreement chart and being authorized by SEER program^13^, the username and password were assigned for SEER*Stat software login. In the SEER 18 registries dataset, we included patients with primary labeled site pituitary gland (C75.1 only) tumors and the selection criteria were not limited to malignant behavior. Patients with multiple primary sites, non-pituitary adenoma diagnosis, death certificate reporting sources, or diagnosed before 2004 were excluded, leaving 47,180 patients qualified for the final statistical analysis. Only the tumors with documented metastasis were defined as pituitary carcinomas (PCs) and the classification procedures were abided by the SEER Collaborative Stage Metastasis at Distance principles^14^. The tumors with malignant or borderline histologic behavior but without distant metastasis were classified as uncertain behavior pituitary adenomas (UPAs).

### Variables classification

Demographic information was extracted and stratified by age, gender, race, insurance, marital status, urbanization, purchased/referred care delivery area (PRCDA) regions, tumor size, Third Edition of the International Classification of Diseases for Oncology (ICD-O-3), treatment modalities, survival months, and vital status. Age at diagnosis was categorized with a five years interval from birth to 85 + years. The standardized incidence rate was calculated per 100,000 person-years by dividing the number of patients by the observation years and the corresponding population. The general US population and age-adjusted reference population were accessed through the SEER standard population dataset, which was originally obtained from the US Census Bureau's Population Projections Program^15^. Subtotal resection (STR) included local tumor destruction (surgical code 10), local tumor excision (code 20), excisional biopsy (code 27), and partial removal of the primary site (code 30). Gross total resection (GTR) included total surgical removal of the primary site and radical surgery (surgical code 40 and 60, respectively).

### Survival parameters

The survival months and vital status are available in the SEER database and those cases without active follow-up were excluded from survival analysis. The survival proportion curves included tumor subtypes, age groups (≤ 18 years, 19–64 years, and ≥ 65 years), and tumor size (cutoff at the median value: ≤ 16 mm and > 16 mm) and they were obtained using the Kaplan–Meier method and compared by log-rank test. The relevant covariates’ effect on overall survival outcome was submitted to multivariable regression models.

### Statistical analysis

Data were extracted with SEER*Stat statistical software (Version 8.3.8, National Cancer Institute, Maryland, USA). We compared the RC list categorical variables with Person’s Chi-squared test and the normally distributed continued variables with independent sample ANOVA. When the data showed an abnormal distribution or heterogeneity of variance, the Kruskal–Willis H test was adopted for nonparametric comparison. Cox proportional hazard regression and binary logistic regression were used for multivariate analysis. Two-sided *P* values < 0.05 were considered to be statistically significant. We used SPSS program (Version 25.0, IBM Corp., Armonk, New York, USA) and Prism 8 (GraphPad Software Inc., San Diego, CA, USA) to analyze data and generate illustrations. All the data and statistical results were independently cross-checked by two authors (C.C.) and (Y.H.).

### Ethics approval

As data acquisition in the SEER database is de-identified and poses no risk for individual participants, our study was exempt from review by the ethics committee of West China Hospital.

## Results

### Demographics

From 2004 to 2016, a total of 47,180 pituitary tumor patients with a mean follow-up of 5.1 years were reported in the SEER 18 registries database. Stratified by biological behavior subtypes, 47,030 tumors were classified as typical adenomas, 111 as UPAs, and 39 as PCs. The demographic and clinical characteristics univariate comparisons are presented in Table 1. Approximately three-quarters of the patients were Caucasian and 73% were aged between 19 and 64 years. Among the 4,242 (9%) other race patients, 80% were Asiatic ethnicity. Typical adenoma patients were younger at diagnosis compared to UPA patients (49.4 ± 18.4 years vs. 55.4 ± 16.7 years, *P* = 0.001). PCs accounted for only 0.08% of all pituitary tumors and were more likely to be found in males than typical adenomas (67% vs. 44%, *P* = 0.004). Both UPAs and PCs were significantly larger than typical adenomas in terms of tumor size (*P* < 0.001).

**Table 1:** Demographic and clinical characteristics of patients included in the present study.

Variables^a^	Typical adenomas	UPAs	PCs	*P*
Total patients	47,030	111	39	
Age at diagnosis, mean ± SD, year	49.4 ± 18.4	55.4 ± 16.7	50.9 ± 18.6	0.001**
**Age groups, year**				0.181
≤ 18	1821 (4)	2 (2)	3 (8)	
19–64	34,165 (73)	75 (68)	25 (64)	
≥ 65	11,044 (23)	34 (30)	11 (28)	
**Sex**				0.004*
Male	20,583 (44)	54 (49)	26 (67)	
Female	26,447 (56)	57 (51)	13 (33)	
M:F Ratio	1:1.3	1:1.06	2:1	
**Race**				0.010**
White	33,313 (71)	67 (60)	28 (72)	
Black	8417 (18)	32 (29)	9 (23)	
Other	4228 (9)	12 (11)	2 (5)	
Unknown	1072 (2)	0	0	
**Insurance**^**b**^				0.775
Insured	35,553 (91)	71 (90)		28 (88)
Uninsured	1617 (4)	2 (3)		1 (3)
Unknown	1782 (5)	6 (8)		3 (9)
**Marital status**				0.581
Married	30,682 (65)	73 (66)	22 (56)	
Unmarried	12,750 (27)	32 (29)	13 (33)	
Unknown	3598 (8)	6 (5)	4 (10)	
**Urbanization**				0.309
Urban	42,601 (91)	97 (87)	37 (95)	
Rural	4296 (9)	14 (13)	2 (5)	
Unknown	133 (0.2)	0	0	
**Region**				< 0.001**
Pacific coast	23,428 (50)	37 (33)	13 (33)	
East	17,083 (36)	63 (57)	20 (51)	
Other	6519 (14)	11 (10)	6 (15)	
Tumor size^c^, mean ± SD, cm	18.1 ± 13.2	29.7 ± 18.7	27.6 ± 13.4	< 0.001***

*UPAs* Uncertain behavior pituitary adenomas, *PCs* Pituitary carcinomas.

^a^Data were presented in numbers and percentages unless noted otherwise, unknown status was no included in statistical comparisons.

^b^For cases registered 2007 and onward.

^c^Only cases with exact tumor size recorded were calculated (n = 36,732).

*P value for difference between PCs and typical adenomas, **P value for difference between UPAs and typical adenomas, ***P value for difference between UPAs + PCs and typical adenomas.

Most patients (87%) had a not specified pituitary adenoma (8272/0) histology, while pituitary carcinoma (8272/3, 85%) was the most frequent type in ICD-O-3 malignant designation (Table 2). Overall, 21,340 patients (45%) underwent surgical resection and most of them (94%) received surgery as their sole treatment modality. Additionally, 1676 patients (4%) received radiation therapy, and beam radiation (99%) was the most frequently utilized method. Also, irradiation was more likely to be performed on patients with UPAs and PCs than typical adenomas (*P* < 0.001) (Table 3). It should be noted that UPAs and PCs remained highly consistent in terms of clinical and demographic characteristics, and no significant differences were observed between these two groups.

**Table 2:** Histology and behavior of primary pituitary tumors based on ICD-O-3.

Histologic behaviors and codes	n (%)
**Benign behavior**	
8272/0: Pituitary adenoma, NOS	41,009 (87)
8140/0: Adenoma, NOS	3681 (8)
8271/0: Prolactinoma	1984 (4)
Others^a^	383 (1)
**Borderline behavior**	
8272/1: Pituitary adenoma, borderline malignancy	13 (68)
8140/1: Atypical adenoma	5 (26)
8270/1: Chromophobe adenoma, borderline	1 (5)
**Malignant behavior**	
8272/3: Pituitary carcinoma, NOS	88 (85)
8140/3: Adenocarcinoma, NOS	7 (7)
8246/3: Neuroendocrine carcinoma, NOS	6 (6)
8280/3: Acidophil carcinoma	3 (3)

*NOS* Not otherwise specified.

^a^Included chromophobe adenoma (8270/0), acidophil adenoma (8280/0), basophil adenoma (8300/0), monomorphic adenoma (8146/0), mixed acidophil-basophil adenoma (8281/0), mixed cell adenoma (8323/0), and macrofollicular adenoma (8334/0).

**Table 3:** Treatment strategies of patients with primary pituitary tumors.

Variables	Typical adenomas	UPAs	PCs	*P*
**Surgery**				0.015*
Yes	21,258 (45)	63 (57)	19 (49)	
None/unknown	25,772 (55)	48 (43)	20 (51)	
**Radiation**				< 0.001**
Yes	1647 (4)	21 (19)	8 (21)	
None/unknown	45,383 (96)	90 (81)	31 (79)	

*UPAs* Uncertain behavior pituitary adenomas, *PCs* Pituitary carcinomas.

Data presented as n (%) or just numbers.

*P value for difference between UPAs and typical adenomas. **P value for difference between UPAs + PCs and typical adenomas.

### Incidence

**Figure 1:**
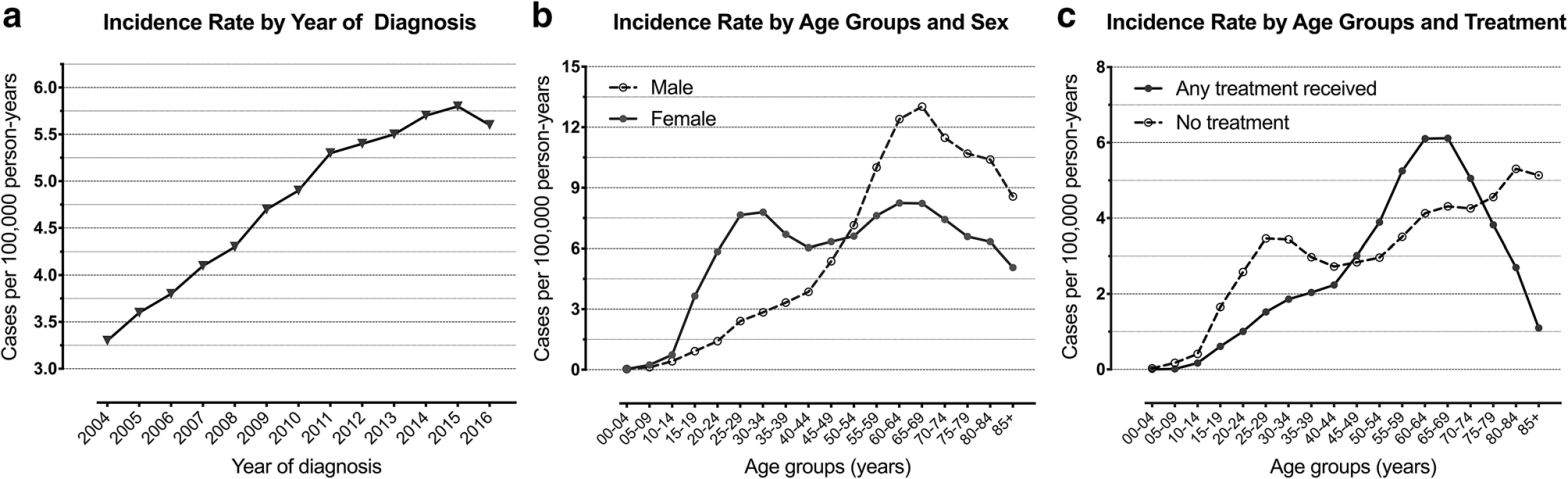
Standardized incidence rate of primary pituitary adenoma by (**a**) year of diagnosis, (**b**) age/sex groups and (**c**) age/treatment groups. Reference population is the general US population or the population in corresponding groups.

The overall standardized incidence rate was 4.8 cases per 100,000 person-years over 13 years of surveillance. Most patients were female in gender (56%), and the incidence rate was higher in female patients than male (5.3 cases per 100,000 person-years vs. 4.3 cases per 100,000 person-years). For the whole population, the annual incidence rate continually trended upwards, with a peak seen in 2015 (5.8 cases per 100,000 person-years) (Fig. 1a). When adjusted for corresponding age groups and sex, a bimodal age-related distribution was observed in female patients, with a first peak seen in adults aged 25–34 years and a second peak in the elderly aged 60–69 years. A unimodal age-related distribution was seen in males and the incidence rate was notably higher in the sixth decade of lifespan (Fig. 1b). When compared the treatment modalities in the incidence rate of pituitary tumors, patients aged 45–74 years were more likely to be treated more actively, otherwise they were probably not to receive any treatment (Fig. 1c).

### Survival

**Figure 2:**
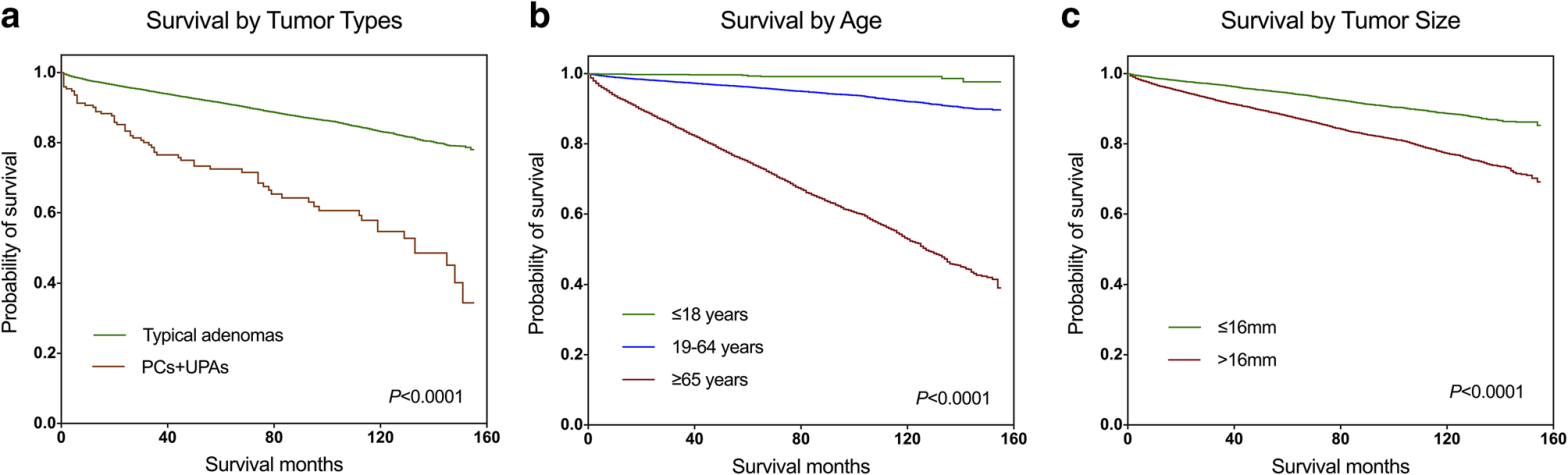
Kaplan–Meier survival curves of all primary pituitary tumors stratified by (**a**) subtypes, (**b**) age groups and (**c**) tumor size. *UPAs* Uncertain behavior pituitary adenomas, *PCs* Pituitary carcinomas.

A total of 46,121 active follow-up patients contributed to 240,459 observation years and the overall survival rates at 3, 5, and 10 years were 94.3%, 91.3%, and 83.1%, respectively. The Kaplan–Meier curves, which compared the difference in survival rate by tumor subtypes, age groups, and tumor size, showed a statistically significant survival advantage for typical adenoma patients compared to UPA and PC patients (Fig. 2a). Also, older age at diagnosis and larger tumor size were associated with significantly worse survival compared to younger patients and patients with relatively smaller tumor sizes (Fig. 2b,c). Table 4 provides the results of multivariate regression analysis for the demographic and clinical variables associated with survival outcome. Females displayed significantly better survival than men with a hazard ratio (HR) of 1.275 (95%CI, 1.203–1.352, *P* < 0.001) and odds ratio (OR) of 1.326 (95%CI, 1.241–1.418, *P* < 0.001). Black patients showed worse survival in both regression models compared to white patients (HR, 1.269 (95%CI 1.185–1.360), *P* < 0.001; OR, 1.339 (95%CI 1.235–1.452), *P* < 0.001), while other ethnic patients had the best overall survival (HR, 0.689 (95%CI 0.615–0.771), *P* < 0.001; OR, 0.627 (95%CI 0.554–0.710), *P* < 0.001). Patients who received radiation treatment experienced worse survival, while the extent of surgical resection was not associated with overall survival in the multivariate analysis (Table 4).

**Table 4:** Results of multivariate regression for demographic and clinical variables associated with overall survival.

Variables^a^	HR (95%CI)	*P*	OR (95%CI)	*P*
**Age groups**				
≤ 18 years	Reference		Reference	
19–64 years	7.883 (4.346–14.300)	< 0.001	7.652 (4.205–13.928)	< 0.001
≥ 65 years	52.266 (28.785–94.903)	< 0.001	59.264 (32.507–108.042)	< 0.001
**Gender**				
Female	Reference		Reference	
Male	1.275 (1.203–1.352)	< 0.001	1.326 (1.241–1.418)	< 0.001
**Race**				
White	Reference		Reference	
Black	1.269 (1.185–1.360)	< 0.001	1.339 (1.235–1.452)	< 0.001
Others	0.689 (0.615–0.771)	< 0.001	0.627 (0.554–0.710)	< 0.001
**Marital status**				
Unmarried	Reference		Reference	
Married	0.987 (0.910–1.071)	0.760	1.078 (0.985–1.180)	0.101
**Insurance**				
Insured	Reference		Reference	
Uninsured	1.259 (1.029–1.541)	0.025	1.246 (1.004–1.547)	0.046
**Tumor size**				
≤ 16 mm	Reference		Reference	
> 16 mm	1.678 (1.557–1.808)	< 0.001	1.801 (1.656–1.960)	< 0.001
**Extent of resection**				
GTR	Reference		Reference	
STR	0.986 (0.874–1.113)	0.823	0.884 (0.770–1.014)	0.079
**Radiation**				
No/unknown	Reference		Reference	
Yes	1.186 (1.047–1.345)	0.008	1.404 (1.207–1.634)	< 0.001
**Subtypes**				
Typical adenomas	Reference		Reference	
UPAs	1.848 (1.303–2.620)	0.001	2.721 (1.696–4.365)	< 0.001
PCs	2.065 (1.026–4.160)	0.042	1.876 (0.786–4.480)	0.057

*HR* Hazard ratio, *OR* Odds ratio, *GTR* Gross total resection, *STR* Subtotal resection, *UPAs* Uncertain behavior pituitary adenomas, *PCs* Pituitary carcinomas.

^a^Unknown data were removed from regression model due to heterogeneity.

## Discussion

The SEER database is widely recognized to be among the most reliable resources in reporting epidemiologic features and survival data for various neoplasms. In this retrospective population-based study, we attempted to address the available information regarding primary pituitary tumors and placed insight into the incidence rate, demographic characteristics, and survival outcome of this group of tumors.

Prior cross-sectional observation studies carried outside the United States identified a standardized incidence rate ranging from 0.6 to 7.4 cases per 100,000 inhabitants per year^6,8–10,16,17^. For the massive population included in the SEER database, an incidence rate of 4.8 per 100,000 person-years that we described is highly generalizable and might be more reflective of the population experience. Additionally, we noticed a bimodal age-related differential distribution in female patients with an evaluated incidence around the third decade of lifespan. This finding is accordant with McDowell et al.^18^ who discovered a higher incidence rate in females during early life and with several other studies which characterized a higher incidence in younger female patients with prolactinomas^8,19,20^. One possible explanation is that clinical manifestations such as infertility, amenorrhea, and galactorrhea would be more pronounced in women of childbearing age, which would increase diagnostic rate^6,8,9^. For prolactinoma being the most frequent tumor subtype reported in previous literature, it is not surprising that females have a higher overall incidence rate than men. In clinical settings, microprolactinomas are approximately twofold more frequent than macroadenomas^21,22^, which contribute to the majority of incidence rate in female patients during the fertile period, not to mention that the true prevalence of microprolactinomas may be underestimated because many suspected tumors are unreported by primary physicians^9^. The effects of prolactinomas on mortality remain controversial, several studies concluded that hyperprolactinemia-related metabolic imbalance is associated with impaired overall survival^23–25^, while others argued that prolactinomas or hyperprolactinemia would not increase mortality rate^26,27^. The hormone-secretion data was, however, missing in the SEER dataset, so we were unable to interpret the specific incidence and survival outcome of each hormonal subtype. Moreover, most patients were vaguely classified as pituitary adenomas in the histopathological designation, which precluded us from inferring the actual prevalence of prolactinoma.

Consistent with previous reports, a constantly increasing annual prevalence has been well characterized in our study, with a nearly doubling of the incidence rate in 2015 compared to 2004. The rising incidence might be related to the significant advancements in neuroimaging, a higher incidentally discovered rate or the increased awareness of pituitary diseases among physicians. Of note, there has been debate about whether the real incidence of pituitary tumor is rising or just the incidentally discovered rate. Raappana et al.^8^ demonstrated in their 16-year period study in Northern Finland that the increase in the incidence rate was caused by incidentaloma rather than symptomatic pituitary adenoma, while Radhakrishnan et al.^28^ discovered that the incidence of symptomatic pituitary tumors also remarkably risen in the Minnesota population. We also found that treatment modalities varied by age group and patients aged > 74 years may not receive any treatment. Since older patients are more vulnerable to surgery-related complications such as hypopituitarism, cerebrospinal fluid leaks, and diabetes insipidus^29,30^, it is understandable that a wait-and-see protocol was more preferable in some cases. Moreover, the first peak of incidence rate in females coincided with the non-treatment group, a possible explanation being that the SEER database does not include data on conventional drug treatment, which would lead to a higher untreated rate.

One year after the study period of current research, the 4th edition of the World Health Organization classification of endocrine tumors has recommended several pathological changes of anterior pituitary gland tumors^31–33^. A major change is the abandonment of hormone-producing adenomas and the subsequent adoption of adenohypophyseal cell lineage as the main principle for classifying pituitary endocrine tumors. This implies that the histological classification based on ICD-O-3 in Table 2 seems to be outdated in the context of new classification. Another notable change is the histological grading of pituitary tumors, which eliminated the controversial term “atypical pituitary adenomas”. And since then, the transitory stage from typically benign adenoma to carcinoma is very vague and shares uneven definition criteria. Current clinical practice in defining aggressive pituitary adenomas (APAs) had been proposed by several groups of experts^34,35^, which is based on the resistance to medical treatments and multiple recurrences despite standard therapies (including surgery, radiotherapy, and chemotherapy). We identified 111 UPAs and 39 PCs in our research and the constituent ratio was consistent with the European Society of Endocrinology’s survey in 2016, which reported 125 APAs and 40 PCs across 17 European countries^36^. PCs are extremely rare types of neoplasms. We reported a prevalence of 0.08% among the entire cohort, which is slightly lower than the 0.1–0.2% found in the existing literature^37–42^. Anthony claimed that 75% of PC cases are diagnosed at autopsy^43^, a lack of autopsy data in our cohort would be the possible explanation for the low prevalence of PCs. Sex predilection is not well established in the literature in terms of PCs, yet, as the two most common types of carcinoma, malignant prolactinoma and corticotropic carcinoma are more likely to be found in men^38,44,45^, which is consistent with the male predominance in our study.

Because of the benign histopathological nature of primary pituitary tumors, the prognosis is excellent as far as overall survival. Nevertheless, pituitary tumors are associated with a substantially decreased overall survival, with a mortality rate two to fivefold higher than that of the general population^4,16,46,47^. The main causes of elevated mortality may be attributed to cardio/cerebrovascular accidents, respiratory diseases, infections, and secondary malignancies^46,48^. These phenomena are speculated to be related to excessive hormone secretion (especially of growth hormone and corticotropic hormone)^2^, hypopituitarism^49,50^, hormone replacement therapy^51–53^, and therapeutic intervention, such as surgery and irradiation^54^.

Not surprisingly, in our study, the Kaplan–Meier survival curves depicted that patients with younger age, smaller tumor size or those diagnosed as typical adenoma experienced improved overall survival. This result was further verified by multivariate regression analysis when we ruled out the effects of relevant covariates. Consistent with several studies^55–57^, we found that female patients displayed a superior survival over men, while some other studies yielded opposite results^16,58^. Still, the gender-related mortality and underlying reasons remain obscure and future matches control studies are warranted to further elucidate this issue. Of interest, the other race patients (mostly Asiatic ethnicity) experienced prolonged overall survival than black/white patients. No existing literature has successfully addressed the relationship between ethnicity and mortality with regard to pituitary tumors. Even though a number of studies have attributed race-related mortality to economic and treatment inequalities in specific disease^59–61^, such disparities in pituitary adenomas merit future investigations. Surgical resection, especially endonasal transsphenoidal surgery, is the current standard of care for primary pituitary tumors except for prolactinoma, which is usually treated with dopamine agonists. When adjusted for sex, age, race, subtypes, and other relevant covariates in the multivariate regression models, GTR, however, still did not yield an improved survival outcome over STR. Ntali et al.^46^ found similar results in their systemic analysis of 546 non-functioning pituitary adenoma cases when they showed that neither extent of removal nor repeated surgery predicted long-term mortality. We also noticed that the patients treated with irradiation tended to have worse survival outcomes either in the entire cohort or in the UPA + PC groups. Hansen et al.^62^ found similar results in their study that included 117 invasive adenomas/pituitary carcinomas in the SEER program from 1973 to 2008. They described no survival advantage for radiation therapy in treating adenomas and speculated that patients with more aggressive tumors would be preferentially offered irradiation. However, because of the biases associated with the unmeasured reasons for receiving or not receiving radiation therapy, any conclusions about its efficacy should be made with caution.

Prior SEER dataset-based studies regarding pituitary tumors focused either on typical adenoma (benign behavior) or on malignant behavior tumors (invasive adenoma and pituitary carcinoma) without jointly analyzing standardized incidence rate and mortality^18,62,63^. The present study found that the annual incidence is increasing and revealed additional factors that associated with overall survival in all histology types with a longer surveillance period. The large sample size of primary pituitary tumors, consensus protocol, and long-term surveillance are the strength of our study; however, our findings should be considered in the context of their limitations. First, the SEER program is subject to the potential for miscoding, which could lead to inaccurate demographic information. Second, the analysis was limited by the available data in the SEER database. As an important topic of benign tumors, recurrence and subsequent treatment data were not provided and therefore prevented us from providing a disease-free survival analysis. Additionally, the hormone secretion function was not provided, as such, the incidence rate and survival of hormonal subtypes could not be calculated. Third, a high proportion of non-operated patients and the resultant low pathological diagnosis rate could contribute to misclassification of pituitary tumors. Finally, information about the quality of life, mental health, and medical comorbidities was not provided, which was critical to assess the burden of the disease.

## Conclusion

In this retrospective SEER-based study, we provide a reliable estimate on the overall standardized incidence rate of patients with primary pituitary tumors and confirm that the annual incidence rate is increasing. More than 80% of the patients survived 10 years and younger age at diagnosis, smaller tumor size, and typical adenoma are associated with prolonged overall survival. Other demographics, such as gender and race, and their association with worse survival outcomes remain to be elucidated in future clinical trials. Although our study provides a comprehensive overview of the incidence, demographics, and survival of patients with pituitary adenoma, the inferential findings should be constrained within the inherent limitations of the SEER database.

## Data Availability

All data are freely available in the SEER datasets.
